# Detection of intact amino acids with a hypervelocity ice grain impact mass spectrometer

**DOI:** 10.1073/pnas.2313447120

**Published:** 2023-12-04

**Authors:** Sally E. Burke, Zachary A. Auvil, Karl A. Hanold, Robert E. Continetti

**Affiliations:** ^a^Department of Chemistry and Biochemistry, University of California San Diego, La Jolla, CA 92093-0340

**Keywords:** hypervelocity, impact ionization, ice grain, astrobiology, mass spectrometer

## Abstract

Astrobiology studies are a top priority in answering one of the most fundamental questions in planetary science: Is there life beyond Earth? Saturn’s icy moon Enceladus is a prime target in the search for life in our solar system, identified by NASA as the second-highest priority site for a flagship mission in the next decade. The orbital sampling technique of impact ionization mass spectrometry indicated the presence of complex organics in the small icy plume particles ejected by Enceladus encountered previously by Cassini. However, high interaction velocities caused ambiguity as to the origin and identity of the organics. Laboratory validation of this technique is needed to show that biosignature molecules can survive an impact at hypervelocity speeds for detection. Here, we present results on the hypervelocity impact of organic-laden submicron ice grains for in situ mass spectrometric characterization with the first technique to accurately replicate this plume sampling scenario: the Hypervelocity Ice Grain Impact Mass Spectrometer. Our results show good agreement with Cassini data at comparable compositions. We show that amino acids entrained in ice grains can be detected intact after impact at speeds up to 4.2 km/s and that salt reduces their detectability, validating the predictions from other model systems. Our results provide a benchmark for this orbital sampling method to successfully detect signs of life and for the interpretation of past and future data. This work has implications not only for a potential Enceladus mission but also for the forthcoming Europa Clipper mission.

## Significance

The search for extraterrestrial life, especially within our solar system, is one of the biggest endeavors of mankind. The icy moons of Saturn and Jupiter, Enceladus and Europa, are particularly promising for hosting life, as they have shown evidence for the three important criteria: water, energy, and organic chemicals. Both moons eject their subsurface ocean material as a plume of icy particles, providing the opportunity to study the ocean composition and potential habitability via plume flythrough sampling. The results reported here provide unambiguous laboratory evidence that we could fly through these plumes at speeds up to 4.2 km/s and successfully detect intact amino acids, an important class of biosignature molecules, in situ with a mass spectrometer.

Whether extraterrestrial life exists is one of the most existential scientific questions to be answered. There are three main conditions for life as we know it: liquid water, a source of energy, and chemical elements that make up the building blocks of life on Earth (1). Some bodies in our solar system show promise for satisfying these conditions (2, 3). For example, the Cassini mission found evidence that a moon of Saturn called Enceladus possesses liquid water in a subsurface ocean (4, 5), a hydrothermal energy source generating cryovolcanic plumes (6, 7), and low- and high-mass organic material (8, 9). Europa, an icy moon of Jupiter, is similarly promising (3). Despite having an ice shell many kilometers thick that would make it difficult to probe the subsurface ocean directly, Enceladus ejects ocean material as a plume of icy particles through cracks in the icy surface at the southern pole (10, 11), forming the E-ring of Saturn (12, 13) and providing the opportunity to perform plume flythrough sampling as a measure of the composition of the subsurface ocean. With this, there is a strong case for a return to Enceladus to search for signs of life (14, 15), either with sample return or with in situ characterization (16, 17).

One effective technique for in situ plume characterization is impact ionization mass spectrometry, which uses the energy of hypervelocity impact (>3 km/s) to vaporize and ionize an incident plume particle for immediate mass spectral analysis (18). The cosmic dust analyzer (CDA) onboard the Cassini spacecraft—as well as the surface dust analyzer onboard the forthcoming Europa Clipper spacecraft—are examples of impact ionization mass spectrometers (19, 20). Analysis of the CDA data from the 0.2 to 2-µm diameter Enceladus plume and E-ring particles showed the presence of three composition categories: pure water-ice grains (Type I), organic-rich grains (Type II), and salt-rich grains (Type III) (13). The Type II grains were found to have varying composition, some containing low-mass volatile organics with concentrations up to tens of millimolar (8), and others containing fragments of high-mass (>200 amu) organics with concentrations up to a percent by mass (9). However, the identification of biosignatures was unclear due to the low mass resolution and upper mass limit of the CDA, as well as high plume interaction velocities (3.5 to 20 km/s) thought to have caused molecular fragmentation of the entrained organics (9, 13).

It is important to validate the capability of impact ionization mass spectrometry to detect specific biosignatures within micron-sized ice grains in the laboratory to support astrobiology investigations (21). This includes the Europa Clipper mission, which may also encounter icy plumes (22). Here, we present results from a laboratory technique that exactly replicates the hypervelocity impact of single ice grains for in situ characterization. Other laboratory measurements performed to date have required significant assumptions regarding how the technique mimics the plume sampling scenario. For example, studies with laser-induced liquid beam ion desorption mass spectrometry (LILBIDMS) have been successful in modeling CDA spectra for peak identification (8, 9, 23–25), but it is uncertain that laser-induced desorption of a liquid water jet is a comparable ionization source to that from the impact of a single solid ice grain. The inverse experiment has been carried out by impacting an amino-acid-laden ice surface with micron-sized iron particles (26), but it is unlikely that this inverse configuration properly mimics the high strain rates experienced by a micron-sized ice grain impacting a metal surface at hypervelocity. Computational modeling of hypervelocity ice grain impacts has also been performed, but these studies are limited to 25-nm diameter grains due to the computational complexity (27, 28). From these studies, it has been concluded that interaction velocities of 4 to 6 km/s would likely satisfy the requirements of efficient impact ionization and minimal molecular fragmentation (27, 29). Here, we report results validating the lower limit using a technique that more accurately replicates the plume sampling scenario. The method allows the mass spectra from single ice grain impacts to be measured as a function of ice grain size and impact velocity up to 4.2 km/s.

## Results and Discussion

**Fig. 1.**
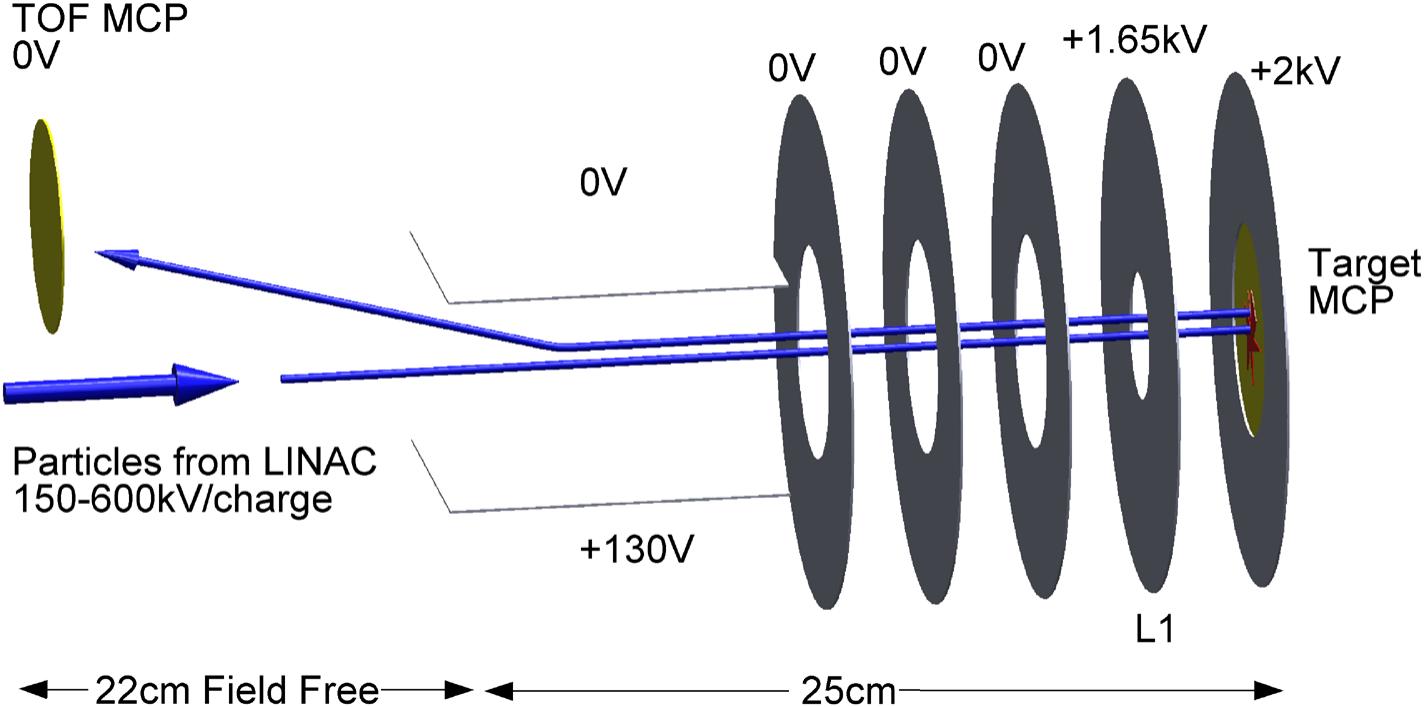
Schematic of the hypervelocity ice grain impact mass spectrometer. Incident particles impact the MCP Target at hypervelocity speeds, and the ions produced by impact ionization are characterized in situ.

Our experimental setup is described in the *Materials and Methods* and briefly consists of the Aerosol Impact Spectrometer (AIS) and the Hypervelocity Ice Grain Impact Spectrometer (HIGIMS). The AIS is used to prepare ~800 nm diameter charged icy particles using electrospray ionization and to accelerate them with a linear accelerator (LINAC) to a controlled final velocity as high as 4.2 km/s (*SI Appendix,* Table S1). A single accelerated particle enters the HIGIMS chamber shown in Fig. 1, where it impacts a microchannel plate (MCP) target, undergoes impact ionization, and the prompt ions are characterized by cation time-of-flight mass spectrometry (TOFMS). The signal from the MCP target marks the time-zero of the mass spectra (*SI Appendix,* Fig. S2). The postimpact mass spectra are recorded for each single-particle impact with known incident velocity (see *SI Appendix,* Fig. S3 for an example of a single-particle spectrum). An average of many single-particle impact spectra is constructed offline. For a detailed characterization of the ice grains prior to impact, refer to *SI Appendix,* Table S1.

### Pure Water-Ice Grains.

**Fig. 2.**
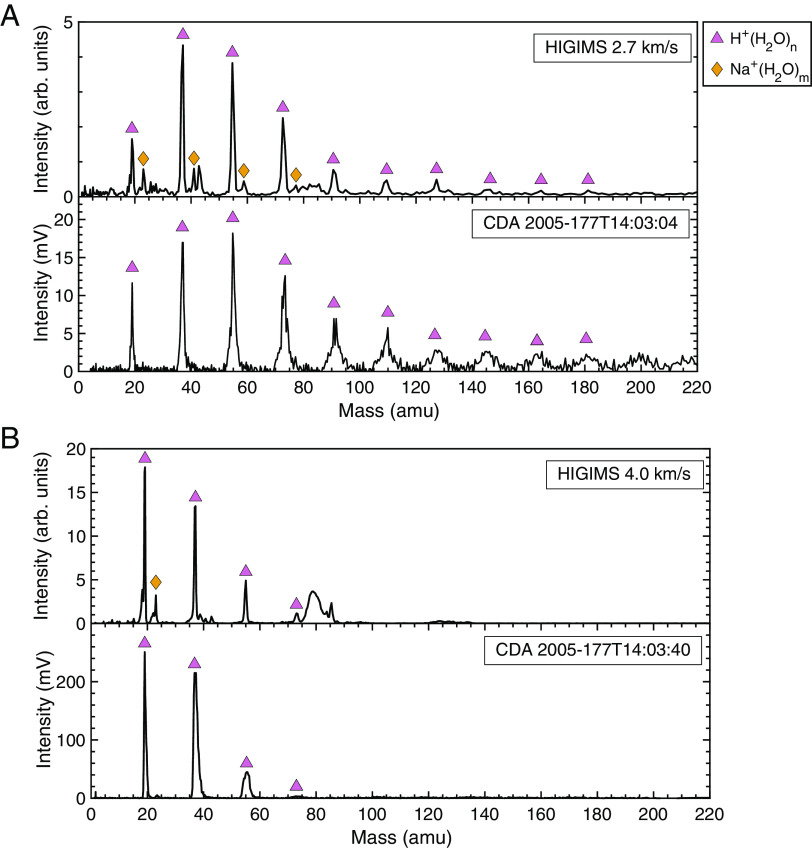
Comparison of pure ice mass spectra collected with the HIGIMS and with Cassini’s CDA. (*A*) Spectra measured at impact speeds of 2.7 km/s show many proton–water cluster peaks, as do some Type I CDA spectra. (*B*) Spectra measured at impact speeds of 4.0 km/s show fewer proton–water cluster peaks but with greater peak intensity, similar to some Type I CDA spectra. While the Type I Enceladus grains are salt-poor by classification, the HIGIMS measurements show the presence of sodium in the samples prepared in the lab.

Measurements with the CDA showed that approximately 65% of the E-ring grains are pure water (Type I) grains (13). Type I grain CDA spectra exhibit a series of proton-water cluster ions H^+^(H_2_O)_n_, some spectra with n ≤ 4 and others with n as high as 15, depending on the energy density of the impact (23). Examples of spectra from the CDA are shown in Fig. 2. As shown in this figure, the mass spectra measured with the HIGIMS match the CDA mass spectra of Type I grains closely. In Fig. 2, impact speeds of 2.7 and 4.0 km/s of ~800 nm diameter pure water-ice grains are shown, and additional impact speed measurements are shown in *SI Appendix,* Fig. S4. The spectra collected as a function of impact velocity display the expected trend: As impact velocity increases, the detectability of H^+^(H_2_O)_n_ with n ≥ 5 decreases. Additionally, the intensity for the smaller clusters increases significantly at higher incident velocities as shown by the relative intensities for the velocity-dependent spectra in Fig. 2 and *SI Appendix,* Fig S4. Spectra collected with an impact velocity of ~2 km/s resulted in no detected proton–water clusters, indicating that this velocity is not energetic enough for impact ionization. This is supported by measurements showing that only ~20% of submicron ice grain impacts exhibit impact ionization behavior at 2 km/s (30). Sodium ion contamination is sometimes present. The good agreement between the CDA Type I grain spectra and those of HIGIMS pure water grains demonstrates the success of this single-particle technique for mimicking the hypervelocity ice impact environment.

A set of unresolved features at ~80 and ~127 amu are observed in most of the spectra, with greater amplitude at higher incident velocity. In some cases, a sharp peak is also present at 85 amu. The identity/source of these features may be ice grains that enter the 10 µm pores on the target MCP, leading to delayed extraction of the ions. Another possibility is ablation of the NiCr glass MCP target material (31), which could produce ionic clusters in those mass ranges with broad distributions of isotopes contributing to the loss in resolution.

### Detection of Amino Acids (AAs) Entrained in Ice Grains.

Organic-rich (Type II) grains comprise 25% of the E-ring grains, making it the second most abundant composition category measured with the CDA (13). The evidence for organics helps make Enceladus promising for future astrobiology investigations, with the goal of clear detection of biosignature molecules. Examples of biosignature molecules inspired by those on Earth include lipids, carbohydrates, peptides, and nucleotides (32). Here, we present the detection of AAs entrained in submicron ice grains using the HIGIMS at impact speeds up to 4.2 km/s. The results validate the conclusions of other analog techniques and models that predicted that these AAs would be detected intact at these incident velocities (29, 33).

AA solutions of 1 mM concentration were prepared in 75:25 H_2_O:MeOH. The methanol was added to facilitate the electrospray of the solutions, and it has no effect on the spectra (see blank spectrum in *SI Appendix,* Fig. S5). For histidine, lower concentrations were measured (*SI Appendix,* Fig. S6), and the histidine molecular ion is easily detectable at concentrations as low as 0.1 mM in the current configuration. Efforts to improve the resolution and sensitivity of the HIGIMS are ongoing in order to demonstrate detection of the estimated low micromolar concentrations of the Enceladus plume grains (34) and ultimately the low nanomolar detection limits typically required for astrobiology investigations (15, 35).

First, AAs that have an amine in their side chain were investigated. Postimpact spectra were collected at incident velocities of approximately 3 and 4 km/s for ~740-nm diameter particles containing histidine, arginine, and lysine individually (see the spectra in *SI Appendix,* Figs. S7–S9). At both impact speeds, the molecular ion of all three AAs was detected, but with higher signal intensity at the lower impact speed. There are also a handful of nonwater peaks at lower masses likely caused by fragmentation of the AA, including peaks in the characteristic mass 29 and/or mass 43 regions observed by the CDA in the low-mass organic Type II grains (8). See *SI Appendix,* Fig. S10 for an example of a Type II CDA spectrum exhibiting these features as compared to spectra from HIGIMS of an AA mixture.

The AA fragment peaks were identified by comparison to electron ionization and collision-induced dissociation spectra from the NIST web book (36). At the impact speeds measured here, the impact ionization appears to be an intermediate ionization source compared to EI and CID, with spectra showing only some of the fragments generated in the hard EI source and showing more fragments than the softer CID source. As shown in *SI Appendix,* Figs. S7–S9, as impact velocity increases, the amplitude of the molecular ion peak decreases and the amplitude of the fragment peaks increases, indicating that the additional impact energy contributes to molecular fragmentation of the AA. In addition to fragmentation, chemical reactions such as the formation of dipeptides may occur upon impact, however no significant peaks were observed in the HIGIMS spectra at masses higher than the molecular ion peak.

**Fig. 3.**
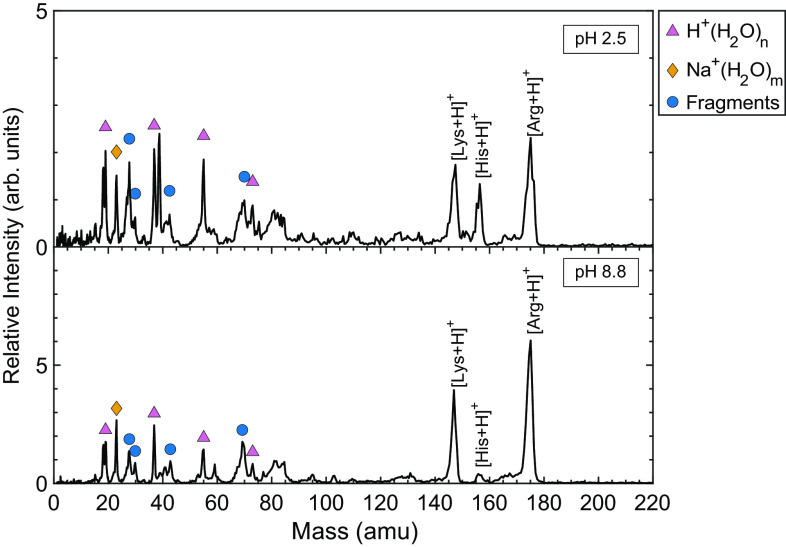
The effect of pKa on the detection limit of an AA at 3 km/s impact velocity. At pH 8.8, lysine and arginine are easily detected, along with a handful of fragment peaks. Histidine is a neutral zwitterion at pH 8.8 and it is less easily detected, but when the pH is lowered to 2.5 the histidine peak is observed. The pH 8.8 and 2.5 spectra are each an average of 230 and 214 single-particle impact spectra, respectively.

The acid dissociation constant pK_a_ is a critical variable for detection of AAs at the current detection limit of the HIGIMS. The positively charged AAs histidine, arginine, and lysine are easily detected in cation spectra, but consider scenarios where the pH of the solution is higher than the pKa of the AA. For example, a mixture containing 1 mM of each AA (His, Arg, Lys), with a measured pH of 8.8, was studied. The postimpact spectra still display Arg and Lys molecular ion peaks, but the His molecular ion peak is greatly diminished (Fig. 3). This is because histidine, with a pK_a_ of ~6, is a neutral zwitterion at pH 8.8. The zwitterion less efficiently forms a cation upon impact than the already positively charged species do. When the AA mixture was acidified to pH 2.5, the histidine molecular ion peak was detected once again (Fig. 3). This effect of pK_a_ on the detection limit of AAs was also observed with experiments using the LILBIDMS (29).

### The Effect of Salt on the Detection of Histidine.

Trace sodium is sometimes seen in the CDA Type I spectra, and sodium and sodium-water cluster peaks appear in most of the organic-rich Type II CDA spectra (9). LILBIDMS experiments show that a salty matrix has an important effect on the detection limit of organic compounds, such that they become more challenging to detect approaching 1 M NaCl due to suppression effects (37). At 0.1 M NaCl, they found that AAs in laser-induced desorption from a salty liquid beam were detected as di- and tri-sodiated adduct cations (AA+45 amu and AA+67 amu) (38). Computational models have shown only minor increases in molecular fragmentation of AAs due to salts with concentrations as high as 2.0 M (28).

**Fig. 4.**
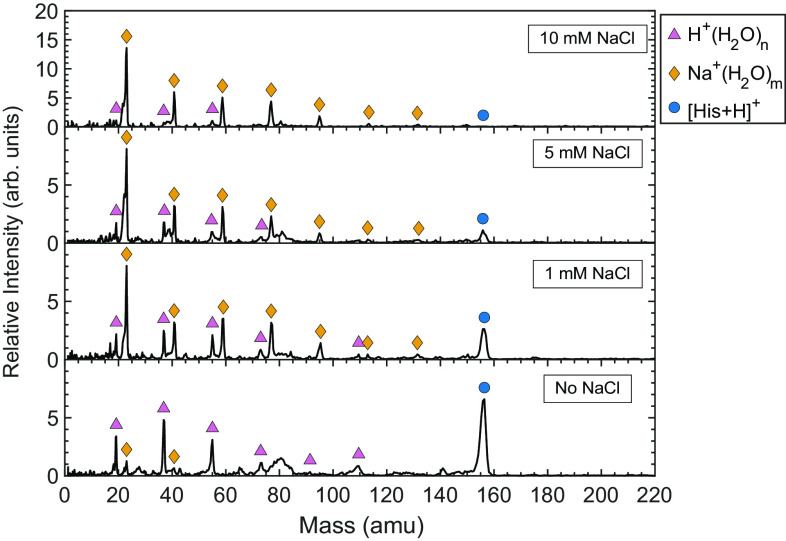
The effect of salt on the detection of 1 mM histidine at 3 km/s impact velocity. As the salt concentration increases, the histidine molecular ion peak diminishes until it is not observed at 10 mM NaCl. No clear fragments of histidine are detected, nor are histidine–sodium clusters. The 0-, 1-, 5-, and 10-mM spectra are each an average of 230, 199, 283, and 117 single spectra, respectively.

With the HIGIMS, a clear effect of NaCl on the detection of histidine was observed. The post-impact spectra of ice grains doped with 1 mM histidine and varying concentrations of NaCl were measured, as shown in Fig. 4. It was observed that as the concentration of NaCl increases, the amplitude of the molecular ion peak of histidine decreases, to the point of not being detected at 10 mM NaCl. While the sodium–water cluster peaks increase in amplitude with increasing salt concentration (thereby diminishing the proton-water cluster peaks), no other sodium clusters were detected. Namely, unlike the LILBIDMS experiment, ions for His+47 amu and His+67 amu are not observed. The pH of all the salty solutions was measured as <5 (*SI Appendix,* Table S1), so the histidine should be in the positively charged state. One hypothesis for the reduced detectability of molecular histidine after impact is that the salt disrupts the hydrogen bond network and the mechanical response of the ice to the impact (28). In that case, we may expect the histidine to be less shielded from the impact energy and thereby undergo molecular fragmentation. However, fragments of histidine (and sodiated fragments) were also not observed in the spectra, so the mechanism for this effect requires further investigation.

The salt concentrations used here (at most ~0.06% by mass) are relatively low in comparison to inferred salt concentrations in some Enceladus ice grains: Type III grains are predicted to have salt concentrations of 0.5 to 2% by mass (24). Given the lower salt concentrations used here, the common features of the Type III spectra [i.e., Na^+^(NaOH) clusters] were not observed.

## Conclusions

These results show that simple AAs survive hypervelocity impacts of single-charged submicron ice grains up to 4.2 km/s. This technique accurately replicates the hypervelocity impact of ice grains on orbital probes for in situ characterization by impact ionization mass spectrometry. The results are consistent with observations from the CDA and the laboratory analog LILBIDMS measurements. Lower impact velocities improve the sensitivity for detection of AAs, but we find that at least ~3 km/s impact speeds are needed for efficient impact ionization. Higher impact velocities still need to be studied to identify the upper-velocity limit for AA detection. Further studies of different biosignature molecules and conditions (i.e., matrix compositions and target materials) may additionally influence the optimal flythrough speed for impact ionization. It was also confirmed that the pK_a_ plays an important role in the detection limit for AAs in ice grain impacts, an important consideration given the predicted basic pH of the Enceladus ocean (24). In addition to planetary science, the HIGIMS technique is relevant to other fields involving particle/surface collisions, including investigation of the effect of particle impacts on substrate surface modification and on sputtering processes, as a variant of secondary-ion mass spectrometry. Future work will include studying different biosignature molecules (including dipeptides, lipids, and nucleotides), testing different target materials, and studying the dependence on ice grain sizes. An exciting direction for these studies will be the search for synthesis of biosignature molecules upon impact, including the formation of AAs and dipeptides as previously observed in aqueous microdroplets (39) and the irradiation of interstellar ice analogs (40, 41).

## Materials and Methods

### The AIS.

The AIS is a submicron particle accelerator (42, 43). A schematic of the AIS with the HIGIMS is shown in the *SI Appendix,* Fig. S1. Particles are generated with electrospray ionization and are transferred into higher vacuum with an aerodynamic lens system that helps to collimate the ion beam (44). A quadrupole deflector is then used to bend a subset of the particles by their kinetic-energy-per-charge ratio. A quadrupole deflector voltage of 200 V was used for all the data presented in this work. According to calculations balancing the evaporative cooling and radiative heating of the particle, it is expected that the droplet freezes to ice in 50 to 200 µs (43). Another model that accounts for nucleation of supercooled droplets predicts freezing on the time scale of 0.65 to 0.85 ms (45). In either case, the droplet is frozen well before it reaches the next stage: characterization of charge, mass-to-charge, and velocity of the ice grain by charge detection mass spectrometry (CDMS) in the nanoparticle electrostatic trap (NET). The NET is a linear trap with an embedded image charge detector (ICD) tube that detects the oscillation of the charged particle between the electrostatic mirrors. With this information, the mass and diameter of the grain can be calculated. The diameter calculation assumes the particle is spherical and has a density equal to that of pure ice (918 kg/m^3^). This density was used for calculating the diameter of all ice grains, even those with entrained organics, which may have a slightly different density. For each particle composition produced for this work, >250 grains were characterized to get the average properties of the particles (*SI Appendix,* Table S1). The average mass-to-charge ratio and initial velocity parameters from the CDMS characterization were used to calculate a generalized LINAC switching timing sequence for each particle composition. With this sequence, individual grains were accelerated through the 41-element LINAC to a controlled final velocity. The potential supplied to the LINAC elements was varied between −7 and −15 kV to achieve different final velocities. The final velocity of the accelerated particle is calculated from the full-width half max of the signal the charged particle induces into a cylindrical ICD at the end of the LINAC (called “ICDL3”). The accelerated particle then enters the HIGIMS chamber.

### The HIGIMS.

The HIGIMS replicates an impact ionization mass spectrometer in space, such that the particle impacts a target, undergoing impact ionization, and the prompt ions are accelerated back towards a detector for time-of-flight mass spectrometric (TOFMS) characterization. A schematic is shown in Fig. 1. The pressure of the HIGIMS chamber is ~1 × 10^−8^ Torr. The accelerated particle enters the chamber and ideally impacts the target, which has a NiCr-plated leaded-glass MCP (Photonis MCP 25/12/10/8 EDR 25 mm diameter, 12-micron center-to-center pitch with 10-micron diameter pores and 8-degree bias angle) detector mounted into the larger target plate. The composition and structure of an MCP are inherently different from the metal plate targets traditionally used in impact ionization mass spectrometers like the CDA, but the use of MCP targets has been previously proposed in the literature (31). The target plate and front face of the target MCP were supplied with +2 kV and the rear of the back plate was floated at +4 kV with a DC to HV DC converter. On the way to the impact target, the ice grain traverses the ion imaging stack electrodes, the last of which (called “L1”) has an inner diameter of 0.550 inch, compared to the 1-inch diameter of the MCP target face. The L1 plate was supplied with +1.65 kV, and the other circular elements of the ion imaging stack were left at ground in this work. The prompt ions from a successful impact with the MCP target are accelerated back toward the LINAC and traverse ~0.5 m to impact the linear time-of-flight detector—a MCP and phosphor screen detector assembly. To steer the particles upwards toward the MCP/phosphor detector, the bottom deflector was supplied with +130 V and the top deflector was grounded. The MCP front face was at ground, the MCP back plate was supplied with +2.70 kV, and the phosphor screen was supplied with +5 kV. In summary, the HIGIMS was optimized for a cation mode, Wiley McLaren focusing arrangement. Optimization was performed by visualizing the phosphor screen with a CCD camera outside the chamber.

Having been accelerated through potentials of 250–615 kV, the incident particles are not deflected by the relatively low potentials of the target and imaging stack electrodes, but owing to beam divergence, occasionally a particle will impact the L1 plate before it can reach the target. The spectra resulting from an L1 impact are gated out of the data presented in this work using the shape of the signal from the charge-sensitive amplifier on the L1 plate. If the L1 signal shows a minimum in a window 2–20 µs *before* the first signal on the MCP target, then the particle passed through the L1 plate and successfully impacted the target (*SI Appendix,* Fig. S2). Spectra associated with all other signal shapes are gated out.

Four signals were amplified, digitized at 50 MHz, and saved for each individual impact: ICDL3, L1 plate, MCP Target, and phosphor screen. The digitizer was triggered off the trailing edge of the accelerated particle peak on ICDL3. A LabView data acquisition code that was capable of sampling ~1,000 data points prior to the trigger was used, allowing for the storage of the ICDL3 particle peak signal. For data sets collected at a final velocity lower than 3 km/s, the data acquisition frequency was reduced to 25 MHz. Several thousand events were measured for each particle composition and velocity combination, and the data were analyzed in MATLAB. The raw data and codes used can be found in the UC San Diego Library Digital Collections (46).

### Data Analysis.

A MATLAB script was used to examine the signals associated with each ice grain impact. For those events where an incident particle was observed to strike the target MCP and generate a detected burst of ions on the linear TOFMS detector, the incident velocity was stored and then time zero for particle impact was determined from the earliest peak on the target MCP. The peaks were identified, set on a common baseline, and summed. A minimum of 100 single-particle spectra was averaged for each spectrum shown in this work. For example, the 2.0, 2.7, 3.1, and 4.0 km/s spectra in *SI Appendix,* Fig. S4 are each an average of 102, 255, 294, and 439 single-particle impact spectra, respectively. A mass calibration was determined empirically from the time of arrival of proton-water cluster ions, sodium-water cluster ions, and the molecular ions of histidine (156 amu), arginine (175 amu), and lysine (147 amu). All spectra shown in this work are plotted with that mass axis calibration. The range over which this calibration can be extrapolated requires further investigation.

### Sample Preparation.

All solutions were prepared using distilled water. The pH of each solution was measured with a SevenExcellence pH meter (Mettler Toledo). Acidified samples were made by adding 1 N HCl dropwise. The bulk solutions were loaded into a 2.5 mL or 5 mL gastight Luer lock glass syringe and delivered to the ESI emitter.

## Supplementary Material

Appendix 01 (PDF)

## Data Availability

Raw and processed HIGIMS data, processed CDMS data, and analysis codes have been made available in the UC San Diego Library Digital Collections (46) and https:/doi.org/10.6075/J0GQ6XXK.
