# Supplementary material for: Detection of intact amino acids with a hypervelocity ice grain impact mass spectrometer
Source: Proc Natl Acad Sci U S A. 2023 Dec 4;120(50):e2313447120. doi: 10.1073/pnas.2313447120 (PMC10723046; doi:10.1073/pnas.2313447120)
Supplement: Supplementary file 1 — Appendix 01 (PDF) [file pnas.2313447120.sapp.pdf]

Supporting Information for

Detection of Intact Amino Acids with a Hypervelocity Ice Grain Impact Mass Spectrometer.

Sally E. Burke, Zachary A. Auvil, Karl A. Hanold, Robert E. Continetti*.

*Robert E. Continetti
Email: rcontinetti@ucsd.edu

This PDF file includes:

Figures associated with the main text regarding methods, control measurements, and additional results, as well as a table containing detailed characterization of the samples/particles prepared.

Contents:

Figures S1 to S10:
- Figure S1. Schematic of the Aerosol Impact Spectrometer.
- Figure S2. Traces from the HIGIMS.
- Figure S3. Example of single particle impact mass spectrum with the HIGIMS.
- Figure S4. Pure water spectra as a function of impact velocity.
- Figure S5. Blank spectrum of 75:25 H_2O:MeOH matrix.
- Figure S6. Histidine spectra as a function of histidine concentration.
- Figure S7. Histidine spectra as a function of impact velocity.
- Figure S8. Arginine spectra as a function of impact velocity.
- Figure S9. Lysine spectra as a function of impact velocity.
- Figure S10. Comparison of HIGIMS amino acid spectra with CDA spectra.

Table S1:
- Table S1. Characteristics of prepared solutions and generated ice grains.

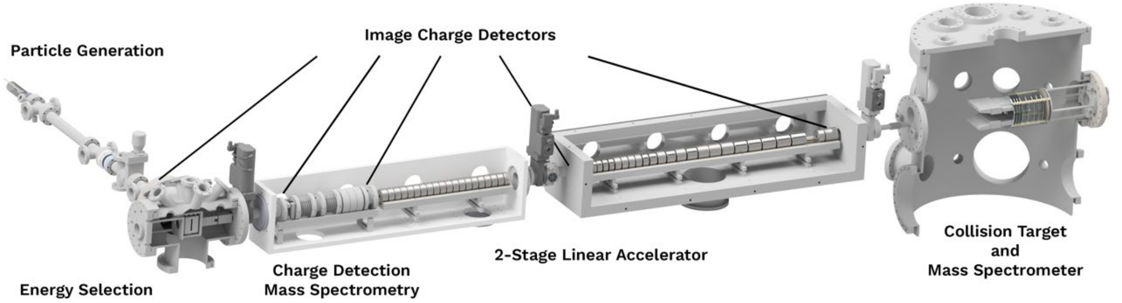

Fig. S1. Schematic of the Aerosol Impact Spectrometer. Charged ice particles are generated with electrospray ionization, transferred into vacuum, energy selected with a quadrupole deflector, and characterized with charge detection mass spectrometry. Using a generalized timing sequence, individual particles are accelerated with a 41-element linear accelerator to a controlled final velocity for impact onto a collision target. The ions from the impact ionization are characterized by mass spectrometry. The trajectory of the charged ice particle is traced through the instrument using cylindrical image charge detectors.

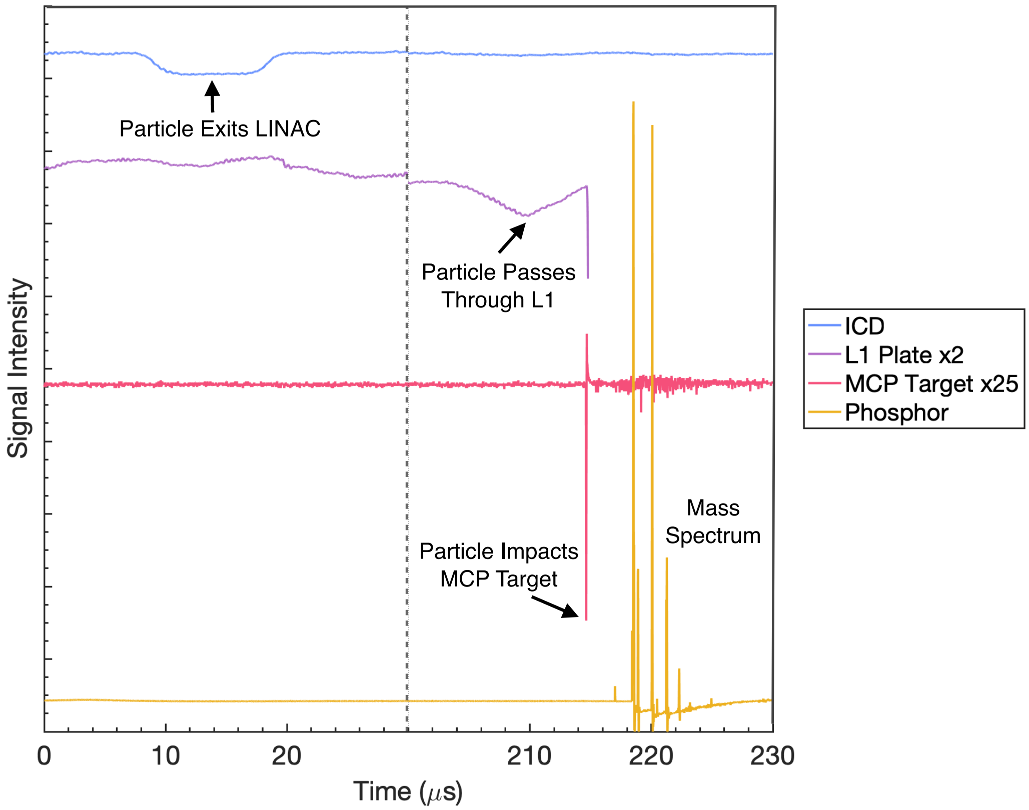

Fig. S2. Traces collected with the AIS and HIGIMS. An accelerated particle exits the linear accelerator and passes through an image charge detector, ICDL3, inducing a square pulse. Some time later, after the particle has traversed the HIGIMS chamber, it passes through the circular electrode directly preceding the target plate, called L1. The peak on the L1 trace is indicative of the particle passing through. Then the particle impacts the MCP Target, producing a sharp peak. The time of this peak is uses as the time-zero for the associated mass spectrum. The impact ionized ions are detected with the TOF mass spectrometer some time later on the phosphor trace.

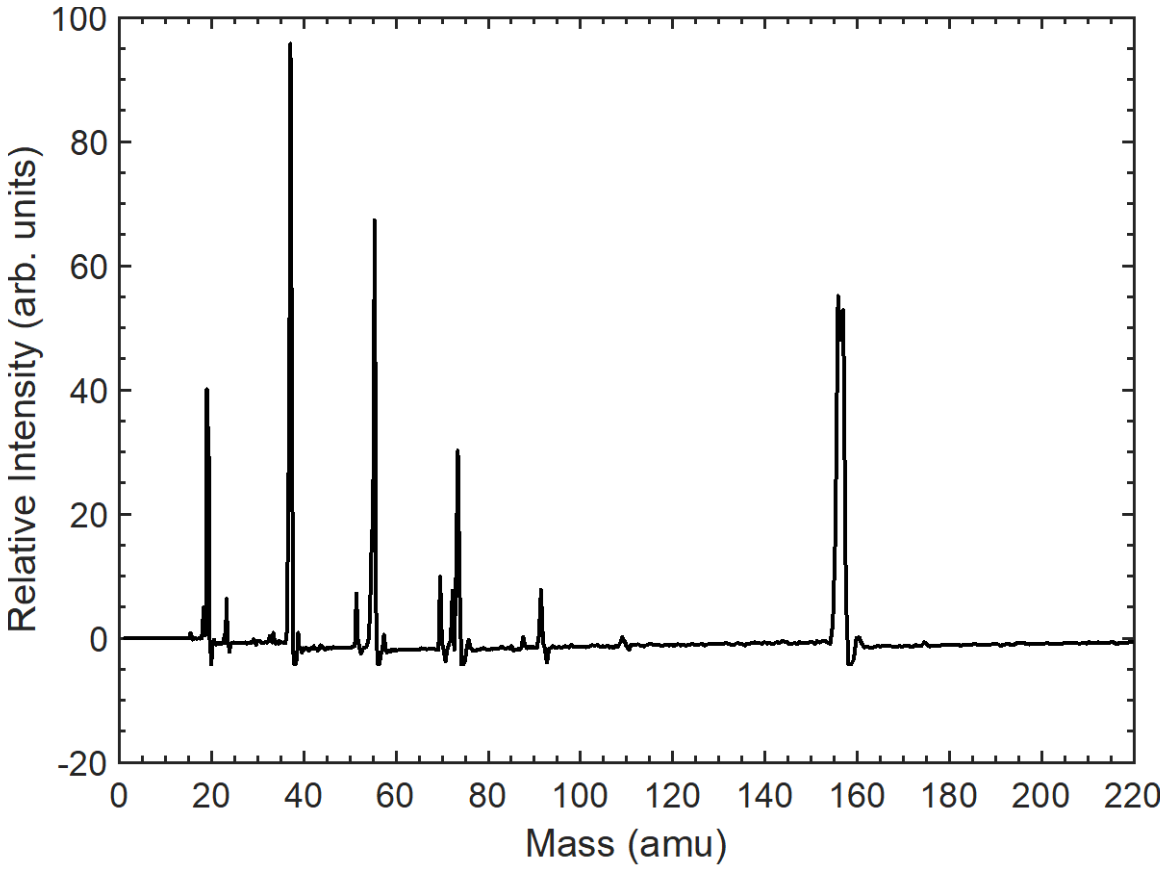

Fig. S3. Example of a HIGIMS spectrum from a single particle impact event. This particle was generated from a solution of 1 mM histidine in 75:25 H_2O:MeOH. Proton-water clusters at mass 19, 37, 55, 73, 91, and 109 are detected. Sodium contamination is detected as the mass 23 Na^+ peak. The molecular ion of histidine at mass 156 amu is also detected. Using the full-width-half-max definition of mass resolution, the resolution of the mass 19 amu peak is ~34 and the resolution of the mass 156 peak is ~81.

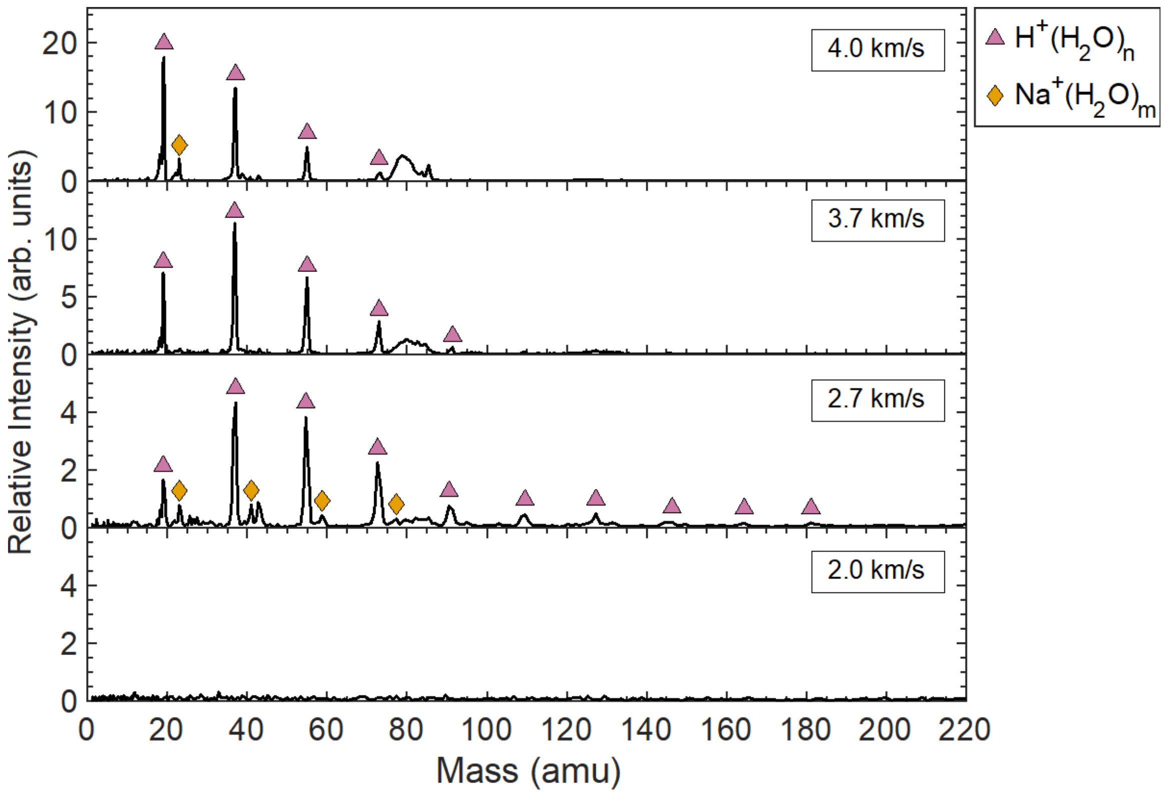

Figure S4. The effect of impact velocity on the HIGIMS spectra of pure water-ice grains. A series of proton-water clusters with n = 1, 2, … are observed. There is increasing formation of the clusters with preference for lower n clusters as velocity increases. Some sodium contamination is detected as m = 0, 1, … sodium-water clusters.

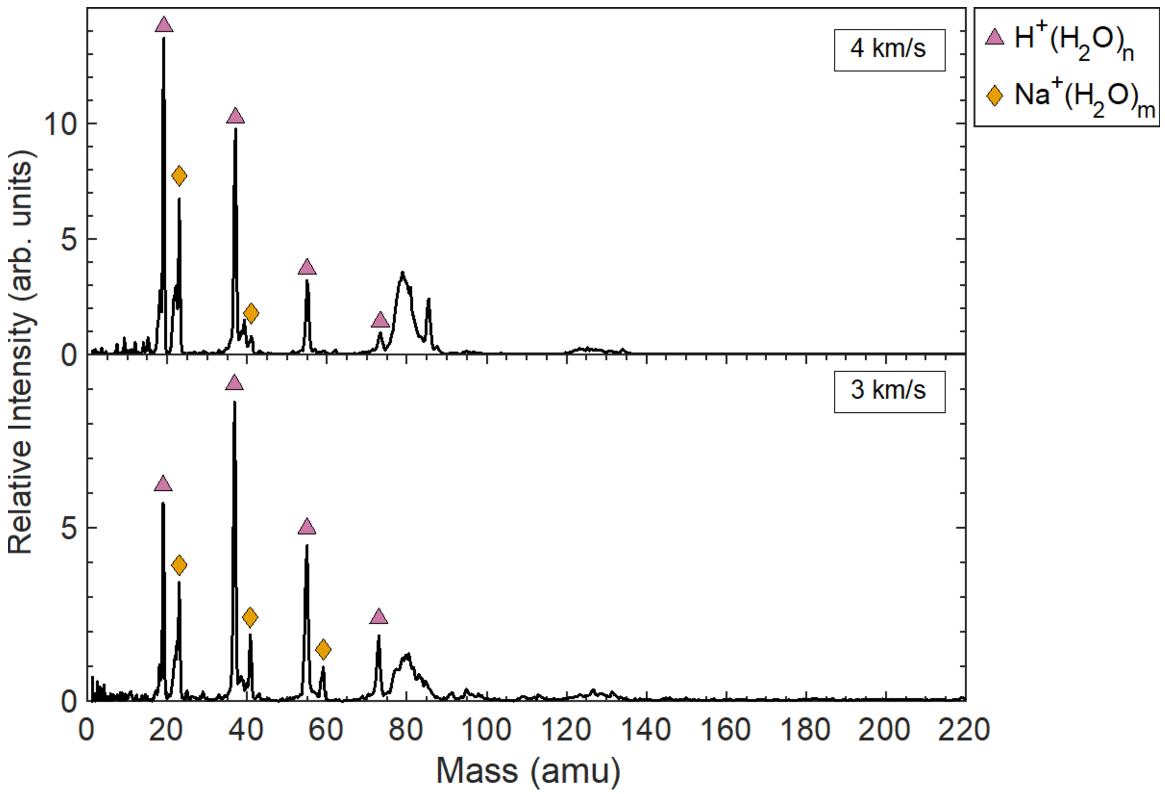

Fig. S5. HIGIMS spectra of 75:25 water-to-methanol blank. The 3 and 4 km/s spectra are each an average of 294 and 344 single particle impact spectra, respectively. No methanol or methanol clusters detected. Some sodium contamination is in evidence from the sodium-water cluster peaks with m = 0,1,….

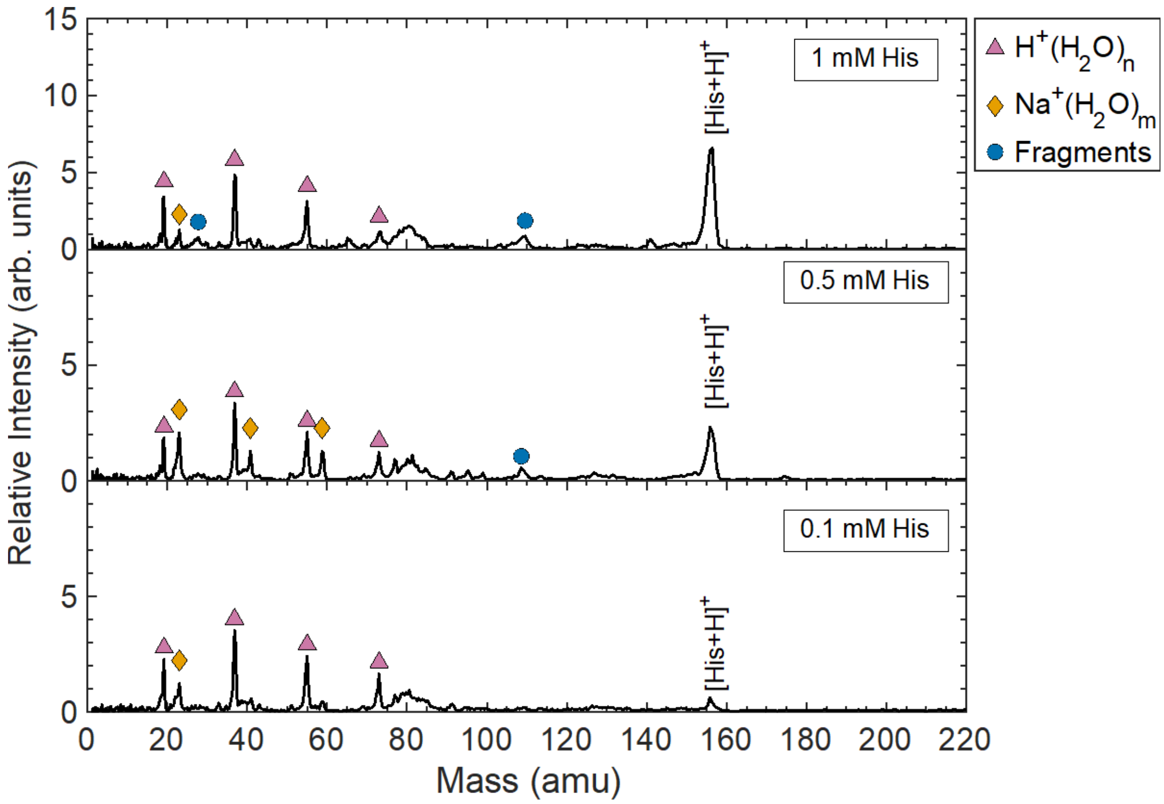

Fig. S6. HIGIMS spectra of various concentrations of histidine in 75:25 water-to-methanol at 3 and 4 km/s. The 0.1, 0.5, and 1 mM spectra are each an average of 270, 242, and 230 single particle impact spectra, respectively. The molecular ion peak is detected, even slightly at 0.1 mM histidine. Other fragment peaks consistent with those on the NIST web book (36) are present at masses 110 and 28 amu.

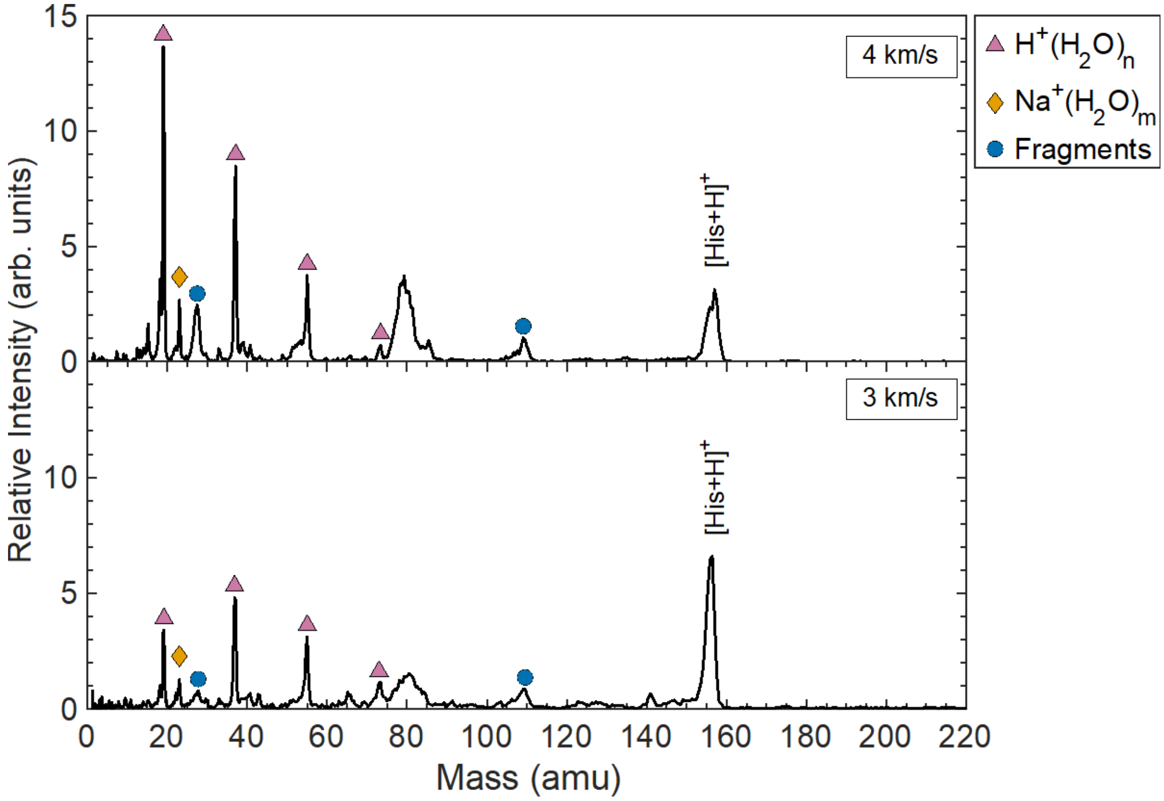

Fig. S7. HIGIMS spectra of 1 mM histidine in 75:25 water-to-methanol at 3 and 4 km/s. The 3 and 4 km/s spectra are each an average of 230 and 269 single particle impact spectra, respectively. The molecular ion peak is easily detected. Several other fragment peaks consistent with those on NIST are present at masses 28 and 110 amu.

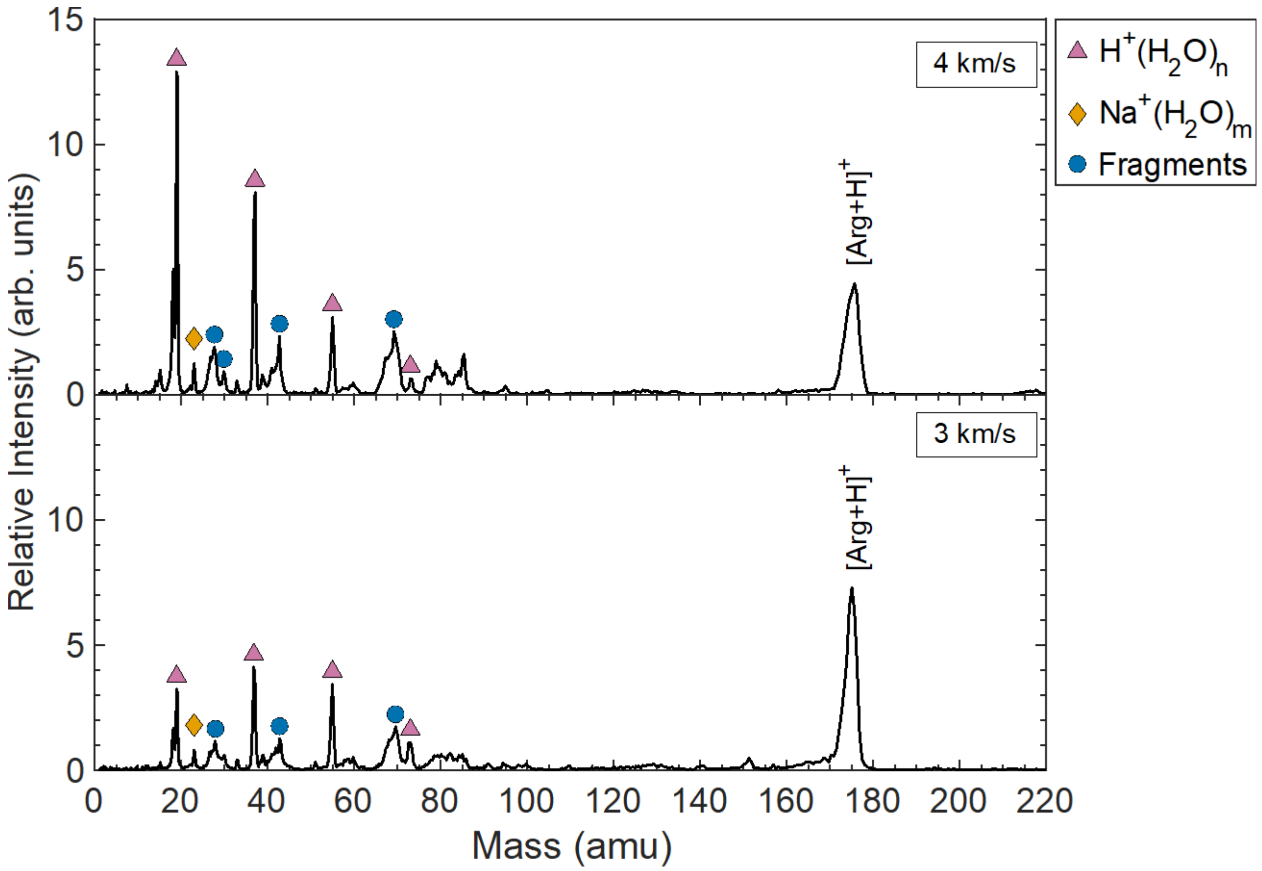

Fig. S8. HIGIMS spectra of 1 mM arginine in 75-to-25 water-to-methanol at 3 and 4 km/s. The 3 and 4 km/s spectra are each an average of 265 and 277 single particle impact spectra, respectively. The molecular ion peak is easily detected. Several other fragment peaks consistent with those on NIST are present at masses 28, 30, 43, and 69 amu.

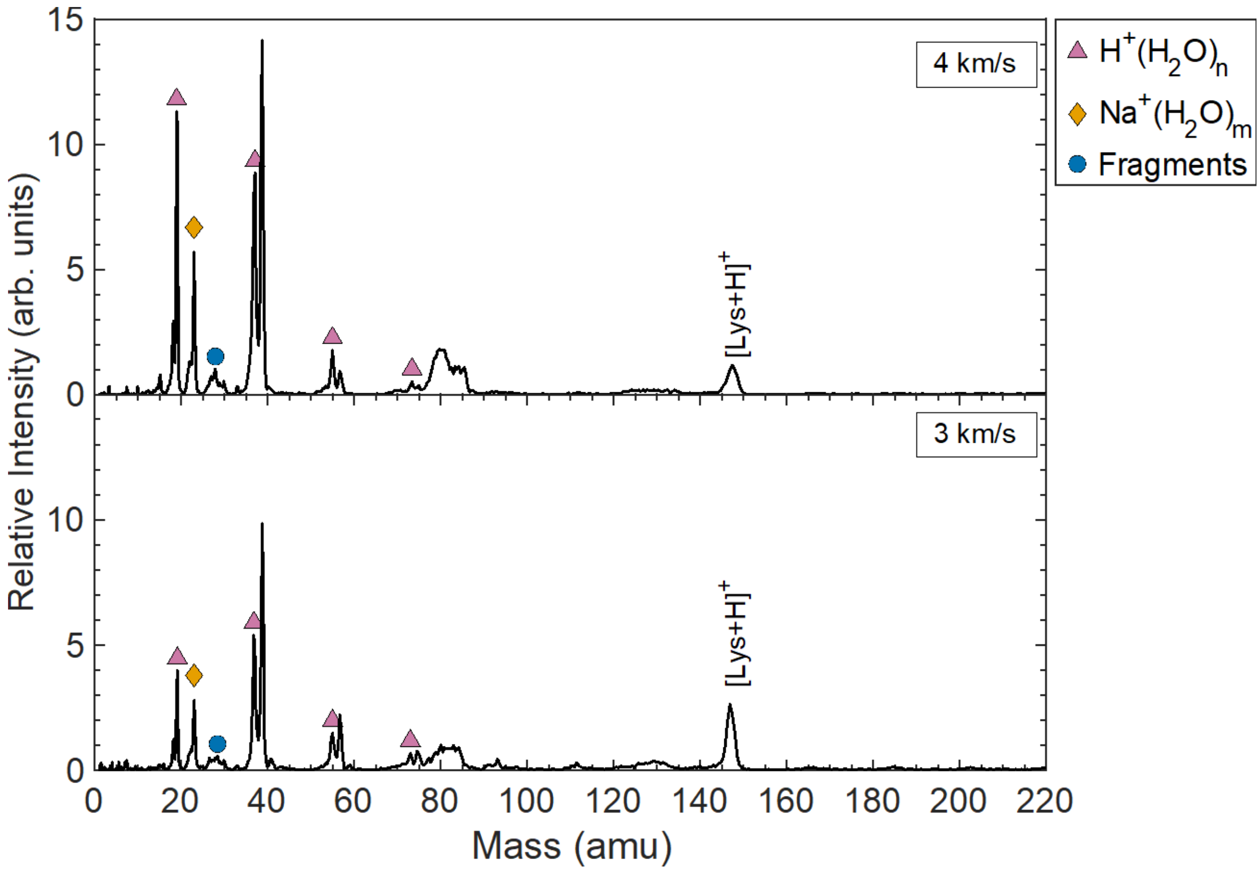

Fig. S9. HIGIMS spectra of 1 mM lysine in 75:25 water-to-methanol at 3 and 4 km/s. The 3 and 4 km/s spectra are each an average of 300 and 299 single particle impact spectra, respectively. The molecular ion peak is easily detected. Several other fragment peaks consistent with those on NIST are present at masses 30 amu. The source of the mass 39 amu peak is unclear.

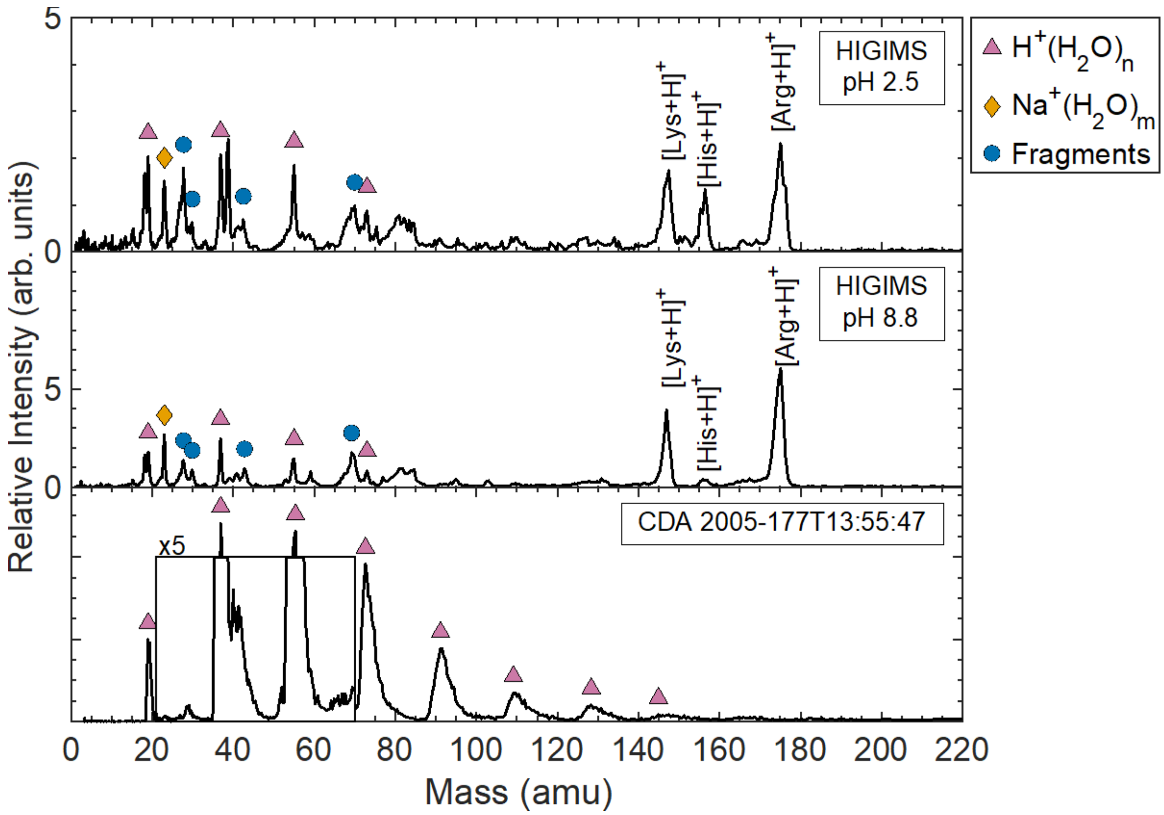

Fig. S10. Comparison of organic-laden ice mass spectra collected with the HIGIMS and with Cassini's CDA. The top two spectra, generated from a solution of histidine, lysine, and arginine (1 mM of each) in 75:25 water-to-methanol, are also shown in Fig. 3. The highlighted regions in the magnified portion of the CDA spectrum show the characteristic features at masses 26-31, 39-45, and 65-68 amu. Fragments at these masses are also observed in the HIGIMS spectra of the amino acid mixture. While molecular ions of organics have not been identified in the CDA data, these fragments are consistent with many organic species, such as the amino acids demonstrated here.

Table S1. Properties of the solutions prepared for the generation of submicron particles to be accelerated. A value of "NR" means the measurement was not taken or not recorded.

Solute	Solvent	Solute Conc. (mM)	NaCl Conc. (mM)	Characterization in the NET Before Acceleration					pH	Impact (Final) Velocity (m/s)
				Initial Vel. (m/s)	Charge (ke)	m/z (g/C)	Mass (pg)	Diameter (nm)		
--	Distilled Water	--	--	82 ± 2	30 ± 5	60 ± 2	0.28 ± 0.04	840 ± 40	5.82	2040 ± 20
										2680 ± 50
										3120 ± 40
										4090 ± 70
--	75:25 H_2O to MeOH	--	--	78 ± 2	23 ± 4	57 ± 2	0.21± 0.03	750 ± 30	2.64	3230 ± 80
										4170 ± 70
Histidine	75:25 H_2O to MeOH	1	10	80 ± 3	20 ±5	58 ± 3	0.19 ± 0.04	730 ± 50	4.97	2960 ± 30
			5	NR	NR	NR	NR	NR	4.99	3140 ± 30
			1	80 ± 3	23 ± 6	57 ± 3	0.21 ± 0.04	750 ± 50	2.77	2910 ± 30
			--	80 ± 2	21 ± 4	60 ± 3	0.20 ± 0.03	750 ± 40	4.41	3100 ± 40
										4030 ± 70
		0.5	--	NR	NR	NR	NR	NR	NR	3000 ± 100
		0.1		NR	NR	NR	NR	NR	NR	3040 ± 30
Arginine	75:25 H_2O to MeOH	1	--	78 ± 2	20 ± 3	58 ± 3	0.19 ± 0.03	730 ± 30	9.70	3150 ± 50
										4040 ± 90
Lysine	75:25 H_2O to MeOH	1	--	78 ± 2	21 ± 4	57 ± 3	0.18 ± 0.03	730 ± 40	10.29	3190 ± 50
										4000 ± 100
Gly-Asp	75:25 H_2O to MeOH	1	--	78 ± 2	22 ± 4	57 ± 3	0.20 ± 0.03	750 ± 40	3.21	3120 ± 50
										4150 ± 90
Glycine	75:25 H_2O to MeOH	1	--	79 ± 2	20 ± 4	57 ± 3	0.18 ± 0.03	720 ± 30	4.37	4170 ± 80
				80 ± 3	15 ± 5	56 ± 4	0.13 ± 0.04	644 ± 70	~1	4200 ± 130
Mixture (His, Arg, Lys)	75:25 H_2O to MeOH	1	--	78 ± 4	21 ± 4	60 ± 3	0.20 ± 0.04	750 ± 40	8.8	3040 ± 30
				79 ± 3	20 ± 5	60 ± 3	0.19 ± 0.04	730 ≠ 60	2.5	3060 ± 30
